# Characterization and pathogenicity of a novel variant infectious bursal disease virus in China

**DOI:** 10.3389/fmicb.2022.1039259

**Published:** 2023-03-17

**Authors:** Yuanling Huang, Gang Shu, Cong Huang, Jingyi Han, Jia Li, Hongjun Chen, Zongyan Chen

**Affiliations:** ^1^College of Veterinary Medicine, Sichuan Agricultural University, Chengdu, China; ^2^Shanghai Veterinary Research Institute, Chinese Academy of Agricultral Science (CAAS), Shanghai, China

**Keywords:** infectious bursal disease virus, novel variant strain, phylogenetic analysis, recombination, pathogenicity

## Abstract

Infectious bursal disease (IBD) is a highly epidemic and immunosuppressive disease of 3- to 6-week-old chicks caused by infectious bursal disease virus (IBDV). Since 2017, there has been a notable increase in the isolation rates of novel variant IBDV strains in China, of which characteristic amino acid residues were different from those of early antigen variants. In this study, one IBDV strain was isolated from a farm with suspected IBD outbreak in Shandong Province, China, which was designated LY21/2. The strain LY21/2 could replicate in MC38 cells with previous culture adaption in SPF chick embryos. Phylogenetic analysis revealed that LY21/2 formed one branch with novel variant IBDVs and shared 96.8–98.6% nucleotide sequence identity with them. Moreover, LY21/2 serving as the major parent underwent the recombination event of a variant strain (19D69), while the minor parent was a very virulent strain (Harbin-1). SPF chicks inoculated with LY21/2 showed no gross clinic symptom, whereas bursal atrophy was exhibited and apoptosis was occurred in 55.21% of bursal cells. The results of histopathology and immunohistochemical staining showed that lymphocyte depletion and connective tissue hyperplasia and IBDV antigen-positive cells were observed in the bursa of LY21/2-infected chicks. Besides, DNA fragmentation was detected in the LY21/2-infected bursal tissue section by TUNEL assay. Collectivtely, these data presented analysis and evaluation of the genetic characteristics and pathogenicity of a novel variant IBDV strain. This study may help in the development of biosafety strategies for the prevention and control of IBDV in poultry.

## Introduction

Infectious bursal disease (IBD) is caused by infectious bursal disease virus (IBDV), which is an immunosuppressive disease of young chicks. IBDV has two serotypes, including serotype I strains which consisted of classic strains, attenuated strains, variant strains, very virulent strains (vvIBDV), and serotype II strains. The genome of IBDV, a member of the family *Birnaviridae* and the genus *Avibirnavirus* (Azad et al., 1985), consists of two segments of double-strained RNA (Muller et al., 1979). The larger fragment A encodes a pVP2-VP3-VP4 polyprotein and a nonstructural protein VP5 in two partially overlapping reading frames, and the smaller fragment B encodes VP1 (Mahgoub et al., 2012). VP2 protein is the major structural protein related to virulence, cell tropism and antigenic variation (Brandt et al., 2001; Coulibaly et al., 2005; Letzel et al., 2007). The antigenic phenotype of IBDV is determined by the hypervariable region (HVR) of VP2 gene (Bayliss et al., 1990; Eterradossi et al., 1997, 1998), in which contains the amino acid (aa) residues 206–350.

In 1957, IBDV was first isolated in the United States (Muller et al., 2003) which was referred to as classic strain, and since then, the virus has spread worldwide and undergone a complex history of evolution. With the emergence of antigenic variant (Jackwood and Saif, 1987) and very virulent IBDVs (Chettle et al., 1989), new challenges on outbreak control and vaccine development have been come as follow. Since IBD first reported in 1979 in southern China (Cui et al., 2013), IBDV mainly of attenuated and very virulent strains infection became prevalent in several provinces and caused huge economic losses (He et al., 2014, 2019). Depending on the virulence, serotype I strains vary in clinical signs and mortality. Some of variant strains may not cause clinical signs or death but do cause bursal lesions. It might account for lack of monitoring and few studies reported about variant IBDVs. However, variant strains were reported and isolated increasingly after outbreaking since 2017 (Fan et al., 2019; Chen et al., 2022; Lian et al., 2022). As is known, IBDV causes immunosuppression mostly by attacking the bursa of Fabricius (BFs) particularly the developing B lymphocytes (Sharma et al., 2000). Moreover, IBDV affects the production of antibodies induced by B cells leading to increased susceptibility to other diseases and vaccination failure (Ranjbar et al., 2019; Fan et al., 2020). Therefore, in consideration of worldwide spread and immunosuppression induced by antigenic variation, it is of significance for preventing and controlling IBD to determine the dominant strains and the critical amino acid residues that may cause immune failure.

In this study, to explore the phenomenon of immunity failure of IBDV vaccine in chicks, we isolated and identified a novel variant IBDV strain (designated LY21/2) from an immunized chick flock with suspected IBD outbreak. The molecular characteristics and pathogenicity were analyzed and evaluated, respectively, by bioinformatics method and by conducting experiment on specific-pathogen-free (SPF) chicks. Our results can provide a reference for epidemic analysis of IBDV in China and studies on immune escape.

## Materials and methods

### Clinical samples

One clinical sample of bursa was collected from broiler in an immunized farm of Linyi, Shandong Province, China. The bursal tissue was homogenized and prepared to 10% (w/v) suspension in phosphate buffered saline (PBS, pH 7.2) with penicillin (100 U/ml) and streptomycin (0.1 mg/ml). The suspension was frozen and thawed three times followed by centrifugation at 10,000 × g for 10 min at 4°C, and then the supernatant was harvested for subsequent detection.

### Animals, cells, and antibodies

SPF chick embryos were purchased from Lihua Agricultural Technology Co., Ltd. (Zhejiang, China) and were incubated at 37°C. Chicks were hatched from the embryos and housed in negative-pressure-filtered air isolators. All the animal experiments were approved by the Shanghai Veterinary Research Institute of the Chinese Academy of Agricultural Sciences and were performed in accordance with the animal ethics guidelines and approved protocols. The mouse colon cancer cell line MC38 was maintained in Dulbecco’s Modified Eagle’s medium (DMEM; BI, Israel) containing 10% foetal bovine serum (FBS; PAN, Germany) and cultured in an incubator at 37°C with 5% CO_2_. Mouse monoclonal antibody against VP2 protein was prepared and stored in our lab. Alexa Fluor 488-conjugated and HRP-conjugated goat anti-mouse IgG antibodies were both stored in our lab (Thermo Fisher Scientific, United States).

### Viral RNA extraction and RT-PCR assay

The total viral RNA was extracted from the bursal sample supernatant using the TRIzol^®^ Reagent (Invitrogen, United States) according to the manufacturer’s instructions. cDNA was reverse transcribed from the viral RNA with M-MLV (H-) Reverse Transcriptase (Vazyme, China) and random primers (Invitrogen, United States). The PCR was performed using a pair of primers, the forward primer (F: 5’-ATGACAAACCTGCAAGATCAAACCCAAC-3′) and the reverse primer (R: 5’-CCTTATGGCCCGGATTATGTCTTTGAAG-3′), which amplified one 1,356-bp fragment of the VP2 gene. A master mix was prepared by using 10 μl of 2 × Taq Master Mix (Vazyme, China), 10 μmol/l of forward primer and reverse primer, 1 μl of cDNA and adding ddH_2_O to a total volume of 20 μl. The PCR was performed at 95°C for 5 min followed by 35 cycles at 95°C for 30 s, 56°C for 30 s and 72°C for 1 min and 15 s, and a final extension at 72°C for 10 min.

### Sequencing and analysis

The RT-PCR product of VP2 was analyzed by 1% agarose gel electrophoresis before being purified with the FastPure^®^ Gel DNA Extraction Mini Kit (Vazyme, China) according to the manufacturer’s instructions. The purified product was cloned into pMD-19 T vector (Takara, Japan) and sequenced by Sangon Biotech Co., Ltd. (Shanghai, China). The complete sequence of VP2 gene was assembled using the DNASTAR software (version 5.0, United States). The alignment and phylogenetic analysis based on the nucleotide or amino acid sequences were performed using the Clustal W program of MEGA X software (version 6.4, New Zealand) and the confidence levels were assessed using 1,000 bootstrap replications. Forty-two sequences of very virulent strains, attenuated strains, classic strains, variant strains, and serotype II strains from GenBank were selected as the reference strains (Table 1). Potential recombination event and breakpoints were identified with RDP4 software (version 4.5, Martin et al., 2015). Further verification was performed with the SimPlot software (version 3.5, Lole et al., 1999).

**Table 1:** Reference IBDV strains used in phylogenetic analysis and the sequence alignment.

Serotypes	Phenotypes	Strains	Origins	Genbank accession no.
I	vvIBDV	OKYM	Japan	D49706
		UK611	United Kingdom	X92760
		Harbin-1	China	EF517528
		YS07	China	FJ695138
		GZ/96	China	AY598356
		T09	Nigeria	AY099456
		KS	Israel	DQ927042
		TASIK	Australia	AF322444
		SDH1	Malaysia	AY323952
		Gx	China	AY444873
		SH95	China	AY134874
		KK1	South Korea	AF165150
	Attenuated	J1C7	Japan	EF646853
		Cu-1 M	Germany	AF362771
		JD1	China	AF321055
		B87	China	DQ202329
		HN04	China	KC109816
		Soroa	Cuba	AF140705
		CEF94	Netherlands	AF194428
		ViBursa G	United States	EU162088
		CT	France	AJ310185
		CU-1	Germany	X16107
	Classical	STC	Canada	D00499
		2,512	United States	DQ355819
		P3009	China	AF109154
		B-SD-LY	China	GQ166970
		Irwin Moulthrop	United States	AY029166
		52/70	United Kingdom	D00869
		Cu-1wt	Germany	AF362747
		W2512	China	MN218126
		IBD17JL01	China	MN604241
	Variant	Variant E	United States	AF133904
		GLS	United States	AY368653
		GZ29112	China	AF051837
		GX-NNZ-11	China	JX134483
		ZD-2018-1	China	MN485882
		SHG352	China	MT179720
		19D69	South Korea	MT550878
		YL160304	China	MZ06661
		variant A	United States	M64285
II		23–82	Germany	AF362773
		OH	Canada	`M66722

### Virus isolation

The supernatant above was inoculated into the chorioallantoic membranes (CAMs) of 9-day-old SPF chick embryos. Six serial embryo passages were performed. The main bodies of dead embryos were homogenized together with the CAMs in sterile PBS to make a suspension, and the debris was spun down by centrifugation at 10,000 × g for 10 min at 4°C. The supernatant was filtered by 0.22-μm membrane and then inoculated into MC38 cells. The cells were harvested by freezing and thawing three times if no cytopathic effect (CPE) shown during 5-day monitoring.

### Indirect immunofluorescence assay

Approximately 1 × 10^6^ MC38 cells seeded on a 6-well plate (Nest Biotechnology, China) were infected with the isolate. The cells were fixed with 4% paraformaldehyde after washing three times with sterile PBS at 24 h post-infection. Cold methanol and 5% non-fat milk were, respectively, used to permeabilize for 15 min at −20°C and block the cells for 1 h at room temperature. Then, the cells were incubated with the mouse anti-VP2 monoclonal antibody and Alexa Fluor 488-conjugated goat anti-mouse IgG secondary antibody. After staining the cellular nuclei with DAPI (Beyotime, China), the images were obtained using an inverted fluorescent microscope.

### Pathogenicity experiment

A total of fourteen 3-week-old SPF chicks were randomly divided into two groups. One group (*n* = 7) was inoculated with the isolated strain by intraperitoneal injection and the other (*n* = 7) was inoculated with sterile PBS as mock-infection. Food and water were provided ad randomly throughout the study. All chicks were observed for 10 days and the clinical symptoms were recorded daily. At 10 d post-infection, bursal tissues of two groups were collected and weighed. The BF:body weight index (BBIX) [BBIX = (BF:body weight ratio)/(BF:body weight ratio in negative group)] was calculated along with the standard deviation. Bursa with a BBIX less than 0.70 was considered as atrophy (Lucio and Hitchner, 1979).

### Flow cytometry

Bursal samples were cut into fragments in cold PBS and the resulting tissue suspensions were passed through 40-mm cell strainers. After washing with cold PBS two times, the cells were suspended in 1 × Binding Buffer to adjust the concentration to 1 × 10^6^ cells/mL. Then, 100 μl resulting cells were transferred to a 5-mL culture tube following by staining with 5 μl annexin-V/FITC (20 μg/ml; Solarbio, China) for 10 min and 5 μl PI (50 μg/ml, Solarbio, China) for 5 min at room temperature in darkness. Finally, 400 μl PBS was added to the stained cells and flow cytometry was preformed within 1 h.

### Histopathology, immunohistochemical, and TUNEL staining

For analysis of light microscopy, the bursal tissue samples fixed with 4% paraformaldehyde were dehydrated in a graded series of ethanol, embedded in paraffin, and sectioned serially at a thickness of 4 μm. The obtained tissue sections were collected on slides and stained with hematoxylin and eosin (H&E). Furthermore, immunohistochemical (IHC) staining was subject to perform by using the mouse anti-VP2 monoclonal antibody. As for detection of DNA fragmentation, the sections were stained with the One Step TUNEL Apoptotic Assay Kit (Beyotime, China) according to the manufacturer’s instructions. Positive cells labeled red fluorescence were detected by inverted fluorescence microscope.

### Statistical analysis

All statistical analyses were performed using GraphPad Prism (version 8.01, United States). Difference in this study was compared by unpaired *t*-test and statistical significance was set at *p* < 0.05. ** represents *p* < 0.01 and **** represents *p* < 0.0001.

## Results

### Isolation and identification of the novel variant IBDV isolate

**Figure 1:**
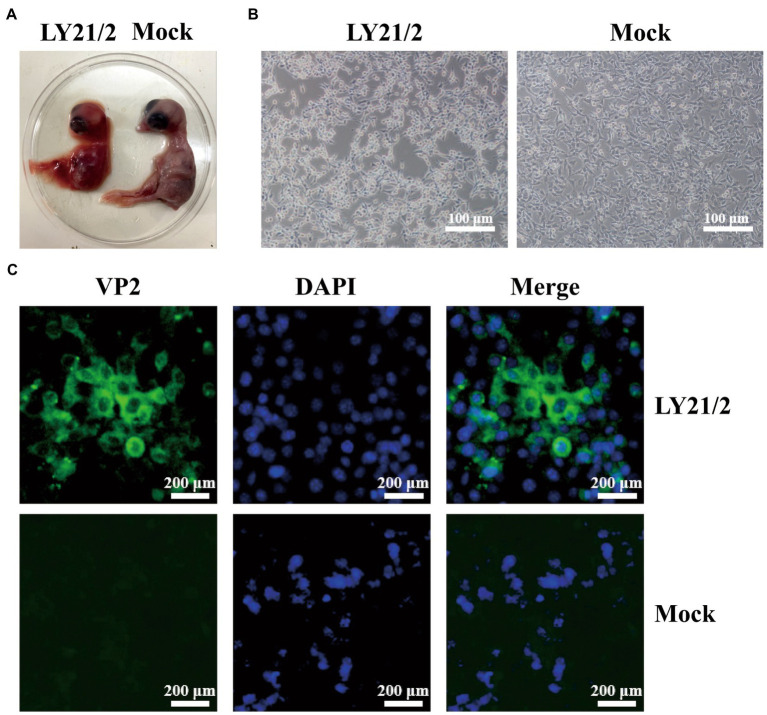
Isolation and identification of the variant IBDV strain LY21/2. **(A)** Isolation of the strain LY21/2 in 9-day-old chick embryo. Dwarfed carcass with congestion and haemorrhage was showed in dead embryo infected with LY21/2. **(B)** Isolation of the strain LY21/2 in cultured MC38 cells. The cells undergone shrinkage and aggregation at 24 h post infected with LY21/2. Scale bar, 100 μm. **(C)** Immunological identification of the strain LY21/2 VP2 proteins expressing in MC38 cells. MC38 cells with LY21/2 infection were fixed and incubated with the mouse anti-VP2 monoclonal antibody followed by an Alexa Fluor 488-conjugated goat anti-mouse IgG secondary antibody. MC38 cells expressing VP2 proteins (green) and nuclear staining (blue) with DAPI were merged. Scale bar, 200 μm.

The bursal sample from suspected IBD outbreak was tested positive for IBDV infection by amplifying a 1,356-bp specific fragment. After detection, the positive supernatant was inoculated into chick embryos for initial culture adaption. Embryos died within 72–120 h after inoculation with the isolate (designated LY21/2). Dead embryos showed lesions, such as incrassated CAMs and dwarfed carcass accompanied by congestion and haemorrhage (Figure 1A). The strain LY21/2 adapted to embryo culture was used to inoculate MC38 cells which attained shrinkage and agglomeration (Figure 1B). And the isolate was further identified by indirect immunofluorescence assay (IFA) using the mouse anti-VP2 monoclonal antibody. VP2 proteins (green) were present in the cytoplasm of LY21/2-infected cells, nevertheless, no positive signals were detected in negative cells (Figure 1C).

### Molecular characteristics of the VP2 gene

**Figure 2:**
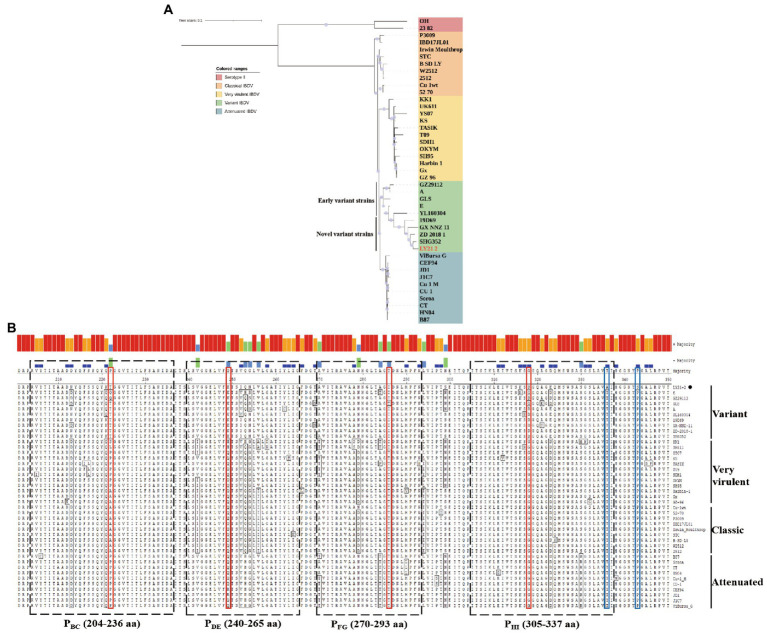
Molecular Characteristics of the VP2 gene. **(A)** Phylogenetic analysis based on the nucleotide sequences of VP2 by MEGA X software v6.4. The phylogenetic tree represented the isolate LY21/2 formed one branch with novel variant IBDVs. The confidence levels were assessed using 1,000 bootstrap replications. The strain LY21/2 is indicated by red font. **(B)** The amino acid sequences in the HVR (206–350 aa) of LY21/2 VP2 were compared with variant strains, classic strains, attenuated strains and very virulent strains. The red boxes represent the characteristic amino acid residues (222 T, 249 K, 286I, and 318D). The blue boxes represent two different amino acid residues (336A and 343S) compared to other reference strains. LY21/2 is shown with a black bot.

A phylogenetic tree based on the nucleotide sequences of the representative fragment of VP2 showed that the IBDV strains were distinctly divided into five major branches, including vvIBDV, attenuated strains, classic strains, variant strains, and serotype II strains. Phylogenetic analysis revealed that the isolate LY21/2 in this study belonged to the same branch as the variant strains (Figure 2A). Moreover, the variant strains can be further divided into two types, the early variant strains and the novel variant strains. The result of further phylogenetic analysis showed that LY21/2 was a novel variant strain (Figure 2A). VP2 gene of LY21/2 showed 93.8–98.6% nucleotide sequence identity with the corresponding regions from variant IBDV reference strains, and 96.8–98.6% identity with the novel variant reference strains. The HVR of VP2 was further analyzed by amino acid alignment, which showed that LY21/2 possessed the same characteristic amino acid residues as the reference novel variant strains, including 222 T, 249 K, 286I, and 318D (Figure 2B). However, two different amino acid residues (336A and 343S) were observed in the HVR of LY21/2 VP2 compared to other reference strains (336 T and 343P; Figure 2B).

### Recombination event of the novel variant IBDV isolate

**Figure 3:**
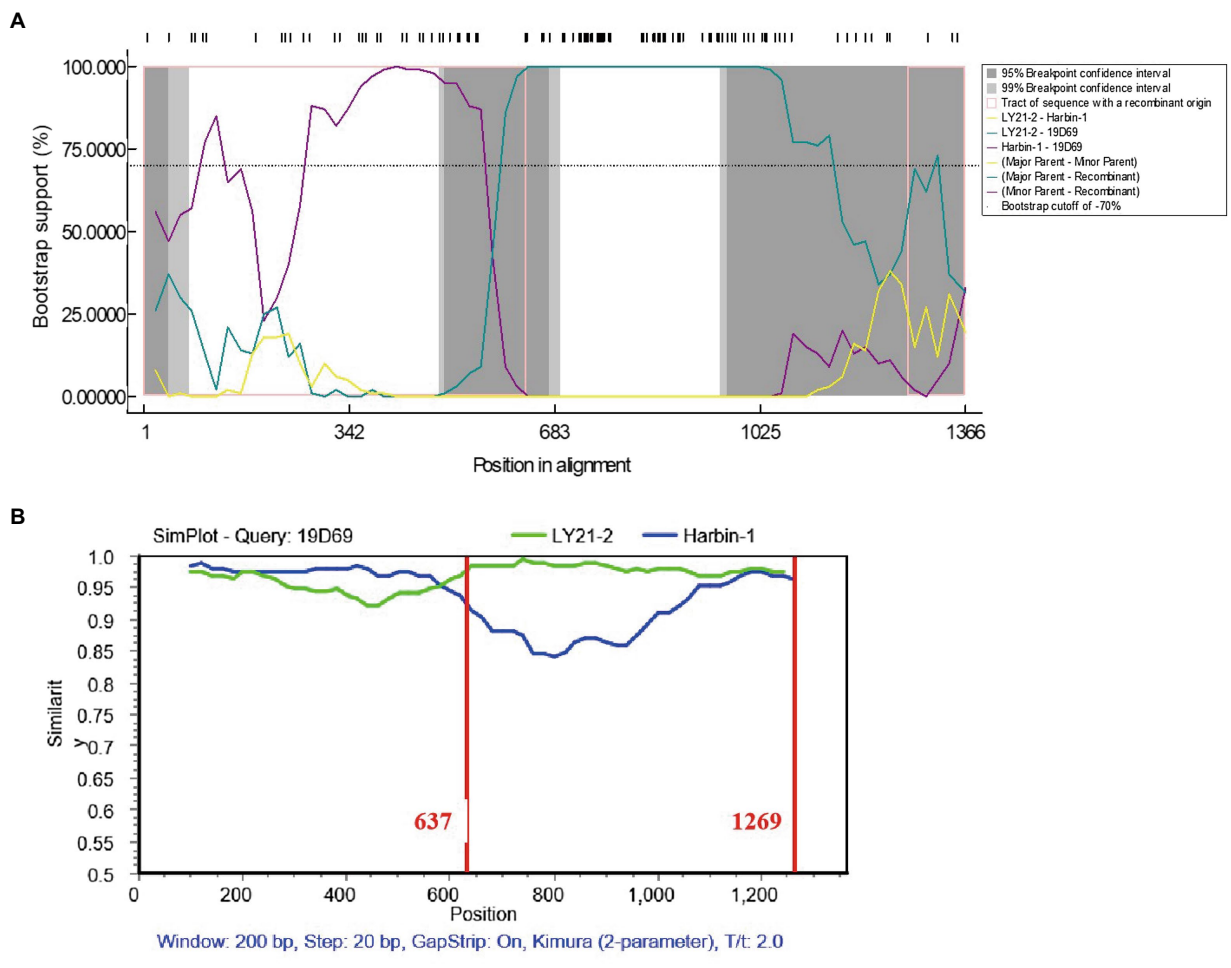
Recombination event of the variant IBDV strain LY21/2. **(A)** Comparisons of the similarity of 19D69 VP2 gene scales with LY21/2 (cyan) and Harbin-1 (purple) were calculated by RDP4 v4.5. The strain LY21/2 was the major parent and the strain Harbin-1 was the minor parent. **(B)** The recombination breakpoints were determined by using Simplot v3.5 and located at nt 637 and nt 1,269, respectively. The major parent (LY21/2) is shown in green, and the minor parent (Harbin-1) is shown in blue. The recombination breakpoints are both exhibited as vertical lines.

Potential recombination events between different IBDV strains were detected using the RDP4 based on VP2 nucleotide sequences. The result revealed that the novel variant IBDV strain LY21/2 as the major parent was involved in a recombination event of the variant 19D69 strain (MT550878, South Korea), and the vvIBDV Harbin-1 strain (EF517528, China) was the minor parent (Figure 3A). SimPlot was used to further confirm the result and to identify the most likely recombination breakpoints. As Figure 3B shown, the most likely recombination breakpoints were at nt 637 and nt 1,269 in VP2 sequence, which were correspond to amino acid residues 213 aa and 423 aa.

### Pathogenicity of the novel variant IBDV isolate

**Figure 4:**
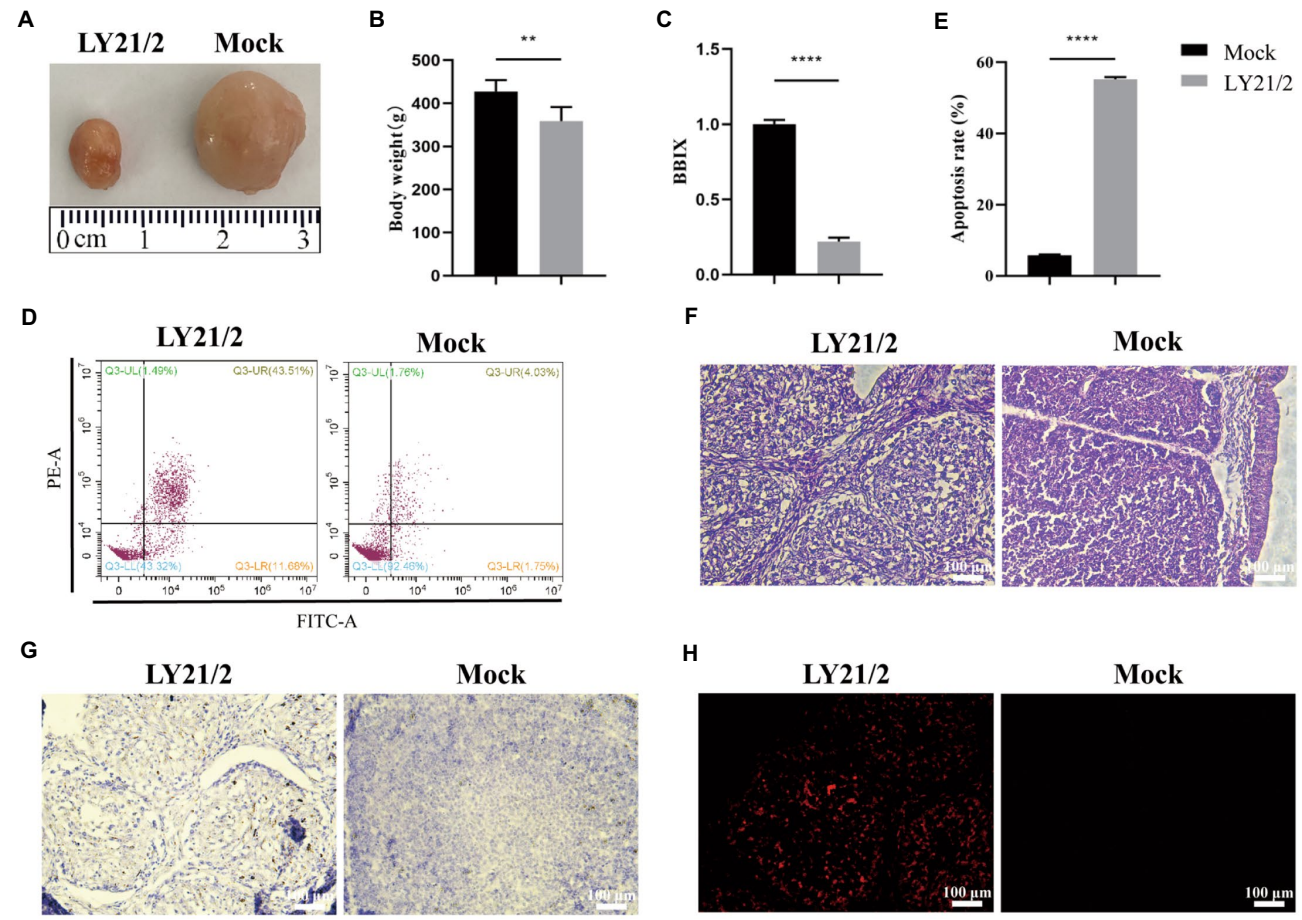
Pathogenicity of the novel variant IBDV strain LY21/2. 3-week-old SPF chicks were inoculated with the IBDV strain LY21/2 and euthanatized at 10 days post-infection. Bursal atrophy was observed in infected chick **(A)**.The mean body weight **(B)** and the BBIX values (BF:body weight index) **(C)** of LY21/2-infected chicks were both negatively influenced. The apoptosis rates of bursal cells of two groups were detected, and the mean rate of LY21/2-infected cells were 49.46% higher than that of mock-infected cells **(D,E)**. **p < 0.01, ****p < 0.0001; an unpaired *t*-test was used to compare differences between the LY21/2-infected and mock-infected group. **(F)** Histological observation of bursal tissues from the mock- and LY21/2-infected chicks. The bursal section of the mock-infected group showed no apparent histological lesions. However, lymphocyte depletion and connective tissue hyperplasia were observed in the bursa of LY21/2-infected chicks. Arrows indicate atrophy of the follicle and lymphocyte depletion in the bursa. Empty triangle indicates a proliferation of connective tissue. **(G)** IHC staining of bursal tissues from the non-infected and infected chicks. No staining was observed in the bursal tissue from non-infected chicks. The bursal sample of LY21/2-infected chicks showed IBDV antigen-positive cells using the anti-VP2 monoclonal antibody. **(H)** TUNEL staining of bursal sections. Infection of LY21/2 induced DNA fragmentation in apoptotic bursal cells (red), whereas, positive signal was hardly detected in normal bursal cells. Scale bar, 100 μm.

To evaluate the pathogenicity of the novel variant IBDV strain LY21/2, 3-week-old chicks were inoculated with LY21/2 or sterile PBS by enterocoelia. Chicks inoculated with LY21/2 showed no gross clinical symptoms or mortality compared to the mock-infected chicks. The body weight of LY21/2-infected chicks was negatively influenced (*p* < 0.01; Figure 4B). Bursal atrophy was observed in LY21/2-infected chicks (Figure 4A), and as expected, the BBIX of infection group (0.219 ± 0.027) was lower than 0.7 (Figure 4C). After separating the bursal tissues into individual cells, the apoptosis rates of two groups were detected by flow cytometry (Figure 4D). Compared with the mock-infection group, the mean apoptosis rate was 49.46% higher in LY21/2-infected group (*p* < 0.0001; Figure 4E). Bursal tissues from the LY21/2-infected group and from the mock-infected group were subjected to pathological sections followed by H&E staining, IHC staining and TUNEL staining. According to the result of H&E staining, the numbers of lymphocytes were decreased and lymphoid nodule atrophy as well as connective tissue hyperplasia were observed in LY21/2-infected bursal tissue (Figure 4F). No obvious pathological changes showed in mock-infected bursal tissue (Figure 4F). IBDV antigen-positive cells were observed in the bursal tissue of LY21/2-infected chicks, while no antigen-positive cells showed in the bursa of mock-infected chicks (Figure 4G). To detect whether DNA fragmentation occurred in apoptotic bursal cells, TUNEL staining was used to perform in bursal tissue sections. Red TUNEL-positive cells were observed in LY21/2-infected bursal tissue, nevertheless, no red signal was detected in mock-infected group (Figure 4H).

## Discussion

After classic IBDV first reported in 1957 (Muller et al., 2003), one antigenic variant IBDV emerged in the 1980s, which led a failure of the immune protection against the classic IBDV (Jackwood and Saif, 1987). In eastern China, it has been reported that subclinical infection of IBD caused by variant strains in some poultry farms since 2017 resulted in considerable economic losses (Coulibaly et al., 2005; Letzel et al., 2007; Fan et al., 2019). Because of the resulting severe bursal damage and immunosuppression, the disease induced by variant IBDV has been regard as an economically significant disease worldwide. It is noticeable that quite a few variant strain cases were detected among very virulent strains, therefore, seasonable monitoring and accurate control are of importance.

IBDV strains can replicate in different systems from avian cell lines such as DF-1 (Chen et al., 2020) or mammalian cell lines such as Vero (Rasool and Hussain, 2006). Nevertheless, the majority of IBDV strains from clinical isolates do not replicate in those systems without adaptation (Soubies et al., 2018; Wang et al., 2020). In addition, there has been an exploration of the possibility of IBDV strains replicating in primary bursal cells (Soubies et al., 2018) or tumor cell lines utilizing the apoptotic characteristics (Delgui et al., 2009). The result of culture in MC38 cells, a kind of tumor cell line from mouse colon, showed that the isolate LY21/2 could achieve virus replication in this cell line for the first time and provided a new reference for *in vitro* systems to replicate IBDV.

As is well-known to all, VP2 is usually used for the identification and analysis of IBDV because of its antigenic determinant (Becht and Muller, 1991; Brandt et al., 2001). In this study, the IBDV strain LY21/2 formed one branch with variant IBDVs according to the phylogenetic analysis based on the VP2 gene nucleotide sequences. We found that LY21/2 shared 93.8–98.6% nucleotide sequence identity with the variant reference stains. The antigenic phenotype of IBDV is determined by the HVR (206–350 aa) of VP2 gene. It was showed by further analysis that the HVR of VP2 gene of LY21/2 contained identical characteristic residues compared to variant IBDVs, including 222 T, 249 K, 286I, and 318D. Besides, the same distinct amino acid sites (221 K, 252I and 299S) were also observed in LY21/2 as the novel variant strains compared to the early variant strains. These characteristic residues were, respectively, located in four loop structures in the HVR which were designated P_BC_ (204–236 aa), PDE (240–265 aa), P_FG_ (270–293 aa) and P_HI_ (305–337 aa; Coulibaly et al., 2005). As reported, the residue 222 T (in P_BC_) is closely related to the antigenicity of virus (Jackwood and Sommer-Wagner, 2011) and replication efficiency (Qi et al., 2016). Besides, residues 249 K (in P_DE_), 286I (in P_FG_) and 318D (in P_HI_) are involved in the reactivity of different monoclonal antibodies or contribute to the antigenic drift of IBDV (Vakharia et al., 1994; Qi et al., 2013; Fan et al., 2022). Nevertheless, previous studies have confirmed that exchange of a single amino acid at the HVR was sufficient for altering the neutralizing properties, replication efficiency or virulence (Qi et al., 2009, 2013, 2016). In this study, we found that one different amino acid residue, T336A, was observed in the HVR of LY21/2 VP2 compared to other reference strains. In spite of that seemed a rare or unique mutation, further experiments are needed to be confirmed.

Homologous recombination contributes to change on antigenic variation and viral pathogenicity. In some previous studies, recombination among classic, very virulent, attenuated and variant IBDV strains in VP2 resulted in the exchange of some antigenic epitopes and change on pathogenicity (Wu et al., 2020; Feng et al., 2022; Guzman et al., 2022). The result of recombination analysis showed that LY21/2 as the major parent was involved in the recombination event of the novel variant 19D69 strain. The recombination breakpoints were at nt 637 and nt 1,269, respectively, corresponding to the residues 213 aa and 423 aa. Notably, the recombination fragment covered the HVR of VP2 hinting that there might be differences in viral antigenicity or pathogenicity after recombination event happened even if they were both classified as the novel variant strains. Moreover, it is still possible of LY21/2 to be a recombination parent of other strains during the process of nature evolution.

Different virulence of IBDV may lead to different pathological changes, such as enlarged and hemorrhagic bursa caused by very virulent strains, while the variant strains cause bursal atrophy. To evaluate the pathogenicity of the novel variant LY21/2 strain, 3-week-old SPF chicks were infected in our study. No gross clinical symptoms or death were present caused by LY21/2 during 10-day observation. However, we weighed all the chicks and found that the mean body weight of IBDV-infected group were negatively influenced. Experiment in which bursectomized chicks survived IBDV infection proves that bursa is the target organ for serotpye I strains (Kaufer and Weiss, 1980). The degree of damage to bursa differs in diverse virulence which has been used as an index of virulence for subclinical cases induced by the variant strains. In this present study, bursal atrophy was observed by necropsy in the LY21/2-infected chicks and correspondingly the BBIX decreased to 0.219 ± 0.027, when it is generally acknowledged that bursal atrophy occurs when the BBIX is less than 0.7. The result of histopathology further verified that after infected with LY21/2, bursal cells depletion was observed in the bursal tissue. Although the novel variant IBDVs do not cause death in chicks, it is likely to weaken the central immune function due to severe atrophy occurred in the bursa which may result in immunosuppression even vaccine failure and secondary or complicating diseases (Tomas et al., 2019; Aliyu et al., 2022). Many factors are involved in tissue or organ atrophy, such as apoptosis. It is reported that IBDV targets the precursors of antibody-producing B lymphocytes in bursa which leads to lymphoid depletion of B cells and apoptosis is responsible for that depletion (Cubas-Gaona et al., 2018). The application of double-staining technique in our study showed that apoptosis in cells of bursa was induced by LY21/2 infection, and correspondingly the apoptosis rate was significantly increased compared to mock-infected group. Besides, DNA fragmentation was detected in bursal tissue of LY21/2-infected chicks which is one of the main features of apoptosis. It might further account for infection by novel variant IBDV strains resulting in bursal atrophy and B lymphocytes depletion in young chicks.

In summary, we isolated and investigated the molecular characteristics and pathogenicity of a novel variant IBDV strain LY21/2. Our results revealed that LY21/2 strain could replicate in MC38 cells, shared a high level of nucleotide sequence identity and several characteristic amino acid residues in the HVR of VP2 gene with the novel variant IBDVs. Besides, LY21/2 as the major parent was involved in a recombination event of other one variant IBDV stain. Interestingly, LY21/2 had no obvious clinical signs in 3-week-old SPF chicks, while it caused bursal atrophy and lesions, as well as increased apoptosis rate. Findings of our study indicate that it is urgent to take action on further molecular analysis and pathogenicity tests for developing a new vaccine program for IBDV.

## Data availability statement

The original contributions presented in the study are publicly available. This data can be found at: https://www.ncbi.nlm.nih.gov/nuccore/OQ566936.

## Ethics statement

The animal study was reviewed and approved by Shanghai Veterinary Research Institute, Chinese Academy of Agricultural Sciences.

## Author contributions

YH and ZC conceived and designed the experiments. YH, CH, HC, and JH performed the experiments. YH, JL, HC, and ZC analyzed the data. HC and GS contributed to reagents/materials/analysis tools. YH, GS, and ZC wrote the manuscript. All authors contributed to the article and approved the submitted version.

## Funding

This work was supported by National Key R&D Program of China (2022YFD1801000).

## Conflict of interest

The authors declare that the research was conducted in the absence of any commercial or financial relationships that could be construed as a potential conflict of interest.

The reviewer KL declared a past collaboration with the authors to the handling editor at the time of review.

## Publisher’s note

All claims expressed in this article are solely those of the authors and do not necessarily represent those of their affiliated organizations, or those of the publisher, the editors and the reviewers. Any product that may be evaluated in this article, or claim that may be made by its manufacturer, is not guaranteed or endorsed by the publisher.
